# Chronic Uridine Administration Induces Fatty Liver and Pre-Diabetic Conditions in Mice

**DOI:** 10.1371/journal.pone.0146994

**Published:** 2016-01-20

**Authors:** Yasuyo Urasaki, Giuseppe Pizzorno, Thuc T. Le

**Affiliations:** 1 Department of Biomedical Sciences, College of Medicine, Roseman University of Health Sciences, 10530 Discovery Drive, Las Vegas, Nevada, 89135, United States of America; 2 Desert Research Institute, 10530 Discovery Drive, Las Vegas, Nevada, 89135, United States of America; Northeast Ohio Medical University, UNITED STATES

## Abstract

Uridine is a pyrimidine nucleoside that exerts restorative functions in tissues under stress. Short-term co-administration of uridine with multiple unrelated drugs prevents drug-induced liver lipid accumulation. Uridine has the ability to modulate liver metabolism; however, the precise mechanism has not been delineated. In this study, long-term effects of uridine on liver metabolism were examined in both HepG2 cell cultures and C57BL/6J mice. We report that uridine administration was associated with O-GlcNAc modification of FOXO1, increased gluconeogenesis, reduced insulin signaling activity, and reduced expression of a liver-specific fatty acid binding protein FABP1. Long-term uridine feeding induced systemic glucose intolerance and severe liver lipid accumulation in mice. Our findings suggest that the therapeutic potentials of uridine should be designed for short-term acute administration.

## Introduction

Uridine is a biologically active pyrimidine with multiple therapeutic potentials. Uridine reduces cytotoxicity on non-cancerous cells due to the administration of anti-cancer drug 5-fluorouracil [1]. Uridine mitigates lipodystrophy associated with the usage of nucleoside reverse transcriptase inhibitors for HIV treatment [2]. Uridine is a nutrient critical for phosphatidylcholine biosynthesis and synapse formation [3]. Uridine improves neurophysiological functions in patients with diabetic neuropathy [4]. Uridine has also been shown to suppress hepatic steatosis induced by the usage of drugs in mice including zalcitabine [5], fenofibrate [6], and tamoxifen [7]. In addition, uridine triacetate (Xuriden), an orally active prodrug of uridine, has recently received an orphan drug designation by the FDA to treat hereditary orotic aciduria.

Uridine has multi-targeted effects because it can be converted rapidly into other biologically active molecules [8]. Uridine is salvaged into pyrimidine nucleotides necessary for RNA and DNA synthesis [9]. Via cytidine triphosphate, uridine promotes membrane phospholipid biosynthesis. Via uridine triphosphate, uridine promotes the formation of uridine diphosphate glucose (UDPG) and uridine diphosphate N-acetylglucosamine (UDP-GlcNAc), which are substrates for glycogen biosynthesis and protein O-linked glycosylation, respectively. Uridine catabolism produces acetyl-CoA, a substrate for protein lysine acetylation. Most interestingly, *de novo* pyrimidine biosynthesis is coupled to mitochondrial respiratory chain [10]. The protective function of uridine on mitochondrial functions is thought to be mediated by its conversion to other pyrimidine intermediates [11].

While the therapeutic potentials of uridine have been well-observed, its side effects on the biological systems have not been fully characterized. Recently, our lab reported that short-term uridine administration induced insulin resistance in the liver of C57BL/6J mice. Our data were consistent with several other independent observations, where a relationship between plasma uridine concentration and systemic insulin resistance was reported in both humans and rodents [12–14]. In this study, we further investigate the effects of long-term uridine supplementation on liver lipid and glucose metabolism.

## Materials and Methods

### Animal models

C57BL/6J mice (male, 10–12 weeks old, Jackson Lab, Bar Harbor, Maine) were divided into four groups: Control mice fed with a lean diet for 5 days (C57BL/6J), mice fed with a lean diet supplemented with uridine for 5 days (C57BL/6J+U), control mice fed with a lean diet for 16 weeks (C57BL/6J+LD), and mice fed with a lean diet supplemented with uridine for 16 weeks (C57BL/6J+LDU). Lean diet was PicoLab Mouse Diet 20 (Cat. No. 5058, LabDiet, Brentwood, MO) that provided 4.6 kcal/g (22% kcal from fat, 23% kcal from protein, and 55% kcal from carbohydrates). For uridine-supplemented diet, uridine was thoroughly mixed with ground pellets at a daily dosage of 400 mg/kg. Mice were not fasted prior to terminal liver tissue collection in early mornings. Liver tissues were collected following the perfusion procedures described previously [15]. All animal studies were performed in conformity with the Public Health Service Policy on Humane Care and Use of Laboratory Animals and with the approval of the Animal Care and Use Committees at Nevada Cancer Institute, Desert Research Institute, and Touro University Nevada.

### Glucose tolerance test

Mice were fasted for 5 hours, then given d-glucose at 0.75 g/kg via intraperitoneal injection. Blood was drawn from the tail vein at 30-minute intervals for two hours after glucose injection and assayed with a glucose meter (Cat. No. 7151G, Bayer, Leverkusen, Germany).

### HepG2 cell cultures

HepG2 cells were cultured in RPMI1640 media (Cat. No. 11875–093, ThermoFisher Scientific, Waltham, MA) with 10% fetal bovine serum, penicillin-streptomycin, and MEM non-essential amino acids (Cat. No. 25-025-Cl, Corning Life Science, Tewksbury, MA). For cells receiving treatments, cells were incubated with 100 μM of uridine and/or 100 μM of PUGNAc for 48 hours prior to the collection of total cell extracts. A deglycosylation enzyme mix (Cat. No. P6039S, New England Biolabs, Ipswich, MA) was employed to reverse the action of uridine in 100 μg of total cell extract. Deglycosylation was performed according to manufacturer’s protocols under non-denaturing condition.

### Identification of FOXO1 glycosylation with 2D Western blots

HepG2 cells were transiently transfected with a plasmid that expresses human FOXO1 with a Myc-DDK or FLAG tag (Cat. No. RC200477, Origene, Rockville, MD) using X-tremeGENE transfection reagent (Cat. No. 06366236001, Roche, Indianapolis, IN). Transfected HepG2 cells were treated with 100 μM uridine for 48 hours, then cell lysates were used for FOXO1 purification using a magnetic DYKDDDDK immunoprecipitation kit (Cat. No. 635694, Clontech, Mountain View, CA). Purified recombinant FOXO1 was evaluated with 1D Western blots using an antibody against Myc-DDK tag or an antibody against O-GlcNAc. Total cell extracts of transfected HepG2 cells were also evaluated with 2D Western blots using an antibody against Myc-DDK tag to examine changes to pI values associated with FOXO1 glycosylation.

### CARS microscopy

A custom-built CARS microscope was employed to examine liver lipid content at CH_2_ vibrational frequency of 2851 cm^-1^ as previously described [16, 17]. Images presented were stacks of approximately 31 frames taken at 1-micron interval along the vertical axis. Liver samples from 9 mice per animal group were examined with CARS microscopy to evaluate liver lipid content.

### 1D Western blot

For total cell extract preparation, HepG2 cells were lysed with RIPA buffer (Cat. No. 89900, ThermoFisher Scientific). For liver tissue extract preparation, homogenization of liver tissues in RIPA buffer was performed. Total cell or tissue extracts were analyzed on 10% SDS-polyacrylamide gel, probed with appropriate primary antibodies and secondary antibodies (Cat. No. 92668070, LI-COR, Lincoln, Nebraska). Immunoblots were detected with the LI-COR’s Odyssey CLx imaging system.

### Primary antibodies

The following primary antibodies were used in this study: O-GlcNAc (Cat. No. cs9875, Cell Signaling, Danvers, MA), FLAG (Cat. No. ab21536, Abcam), Akt (Cat. No. cs9272, Cell Signaling, Danvers, MA), pSer437-Akt (Cat. No. 9271, Cell Signaling), FOXO1 (Cat. No. ab179450, Abcam), pT24-FOXO1 (Cat. No. ab58517, Cell Signaling), PEPCK (Cat. No. sc32879, Santa Cruz Biotechnology, Dallas, TX), MnSOD (Cat. No. 06–984, Millipore, Billerica, MA), FABP1 (Cat. No. ab7847, Abcam), β-actin (Cat. No. cs4970, Cell Signaling).

### 2D Western blot

2D Western blots of HepG2 total protein extracts were performed by Kendrick Laboratories (Madison, WI). A total of 500 μg of total protein extracts or 150 μl was loaded for isoelectric focusing. Carrier ampholine method of isoelectric focusing was carried out in a glass tube of inner diameter 3.3 mm using 2.0% pH 4–8 mix Servalytes (Serva, Heidelberg, Germany). SDS slab gel (10% acrylamide) electrophoresis was carried out for about 5 hours at 25 mA/gel. After slab gel electrophoresis, the gels were transblotted onto a PVDF membrane overnight. The blots were then incubated in primary antibody against the FLAG tag (Cat. No. ab21536, Abcam) overnight. The blots were then placed in secondary antibody (Cat. No. NA931V, GE, Pittsburgh, PA) for two hours, treated with enhance chemiluminescence Western blot detection reagent, and exposed to X-ray film.

### Preparation of liver tissue lysates for capillary isoelectric focusing (cIEF) immunoassays

Approximately 500 mg of frozen liver tissues was added to 300 μl of RIPA buffer (Cat. No. 89900, Life Technologies, Grand Island, NY 14702) containing proteinase and phosphatase inhibitors and homogenized twice at 6 seconds duration. Liver tissue homogenates were incubated on ice for 10 minutes, sonicated 4 times at 5 seconds duration, rotated at 4°C for 2 hours, and centrifuged at 12000 rpm on an Eppendorf 5430R microfuge for 20 minutes at 4°C. Supernatant was collected, prepared in Premix G2 pH 5–8 separation gradient containing pI standards (ProteinSimple), and used for cIEF immunoassays.

### cIEF immunoassays

A NanoPro 1000 cIEF system (Protein Simple, Santa Clara, CA, USA) was employed to profile liver FOXO1 protein glycosylation. Samples of 400-nanoliter volume were separated by isoelectric focusing using the 96-capillary system, followed by immobilization of the proteins onto the inner capillary walls. Primary and secondary antibodies were introduced into the capillaries, followed by chemiluminescence detection reagents. The incubation times were 110 and 55 minutes for primary and secondary antibodies, respectively. Separation time was 50 minutes at 15,000 MicroWatts. All samples were loaded in triplicate into capillaries to evaluate consistency of capillary-to-capillary measurement. Each experiment was done in triplicate to ensure repeatability. A minimum of 9 cIEF measurements were done for each animal group. On average, 40 ng of total cellular protein was loaded into each capillary. Standard exposure time during signal detection was 240 seconds.

### Gluconeogenesis assay

HepG2 cells were cultured to 10^6^ cells per well in a 6-well culture dish in RPMI1640 media (Cat. No. 11875–093, ThermoFisher Scientific, Waltham, MA) with 10% fetal bovine serum, penicillin-streptomycin, and MEM non-essential amino acids (Cat. No. 25-025-Cl, Corning Life Science, Tewksbury, MA). HepG2 cells were washed with 1x phosphate buffer saline for 3 times, replaced with fresh glucose-free DMEM media (Cat. No. A14430-01, ThermoFisher Scientific), and incubated for 4 hours. Then, sodium lactate (20 mM, Cat. No. 7022, Sigma Aldrich, St. Louis, MO), sodium pyruvate (2 mM, Cat. No. 11360–070, ThermoScientific), and uridine (100 μM, Cat. No. U6381, Sigma Aldrich) were added to HepG2 cell cultures and incubated for 8 hours. Cell culture media were collected and analyzed with a commercial glucose assay kit (Cat. No. GAHK20, Sigma Aldrich).

### Statistical evaluation

Data were presented as average value ± standard deviations. Statistical analysis was performed using Excels’ paired Student’s t-test and analysis of variance functions for experimental versus control cell cultures or mice groups. Statistical significance was set at p ≤ 0.05 versus control.

## Results

**Fig 1:**
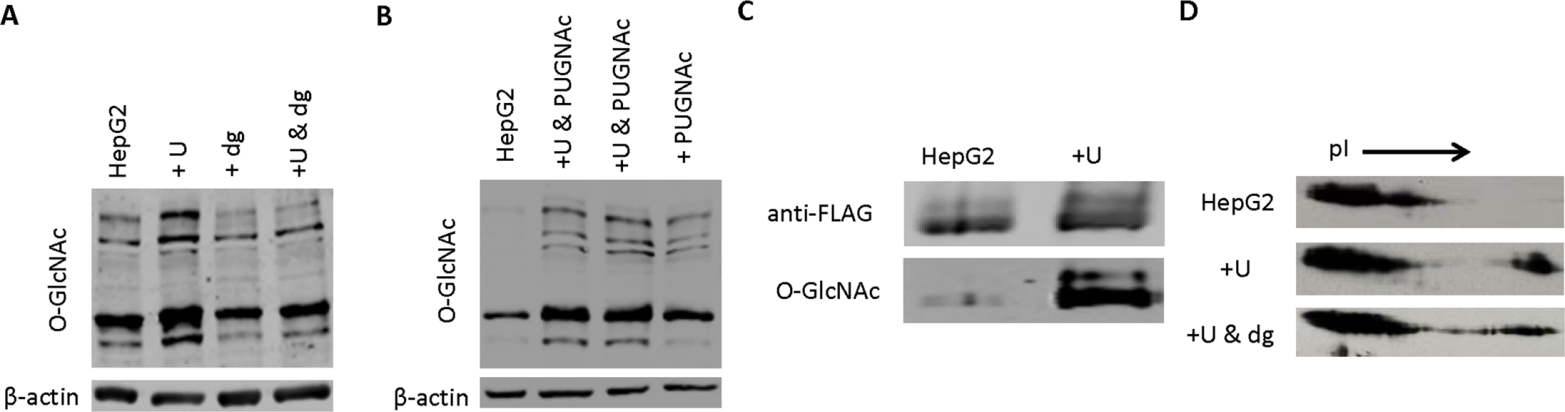
Uridine induces O-GlcNAc glycosylation of FOXO1 in HepG2 cells. (**A**) Antagonistic effects of uridine and deglycosylases on the protein O-linked glycosylation profiles of HepG2 cells. Total cell extracts were evaluated with 1D Western blots using an antibody that recognizes O-GlcNAc. (**B**) Synergistic effects of uridine and deglycosylases inhibitor PUGNAc on protein O-linked glycosylation of HepG2 cells. Total cell extracts were evaluated with 1D Western blots using an antibody that recognizes O-GlcNAc. (**C**) Evidence of uridine-induced O-GlcNAc modification of purified recombinant FOXO1. Immunoprecipitated recombinant FOXO1 protein was evaluated with 1D Western blots using antibodies that recognize FLAG tag (upper panel) and O-GlcNAc (lower panel). (**D**) Antagonistic effects of uridine and deglycosylases on the pI shifts of FOXO1 following O-GlcNAc modification. Total cell extracts were evaluated with 2D Western blots using an antibody that recognizes FLAG-tagged FOXO1.

First, the effects of uridine on protein O-GlcNAc modification was examined in cultured HepG2 cells. Using 1D Western blot and an antibody that recognizes Ser-O-GlcNAc and Thr-O-GlcNAc, total cellular protein from HepG2 cells treated with uridine exhibited a significantly higher protein O-linked glycosylation profile compared to untreated HepG2 cells (**Fig 1A**). Treatment of total cellular protein with a mixture of deglycosylases led to significant reduction in the O-linked glycosylation profiles of both untreated and uridine treated HepG2 cells. The effects of uridine on protein glycosylation of HepG2 cells were further examined with the addition of PUGNAc, an inhibitor of protein O-linked deglycosylation. Addition of PUGNAc increased the overall protein glycosylation profiles of HepG2 cells (**Fig 1B**). Uridine treatment together with PUGNAc further elevated the protein glycosylation profiles of HepG2 cells. Clearly, uridine treatment increased overall protein O-linked glycosylation profiles of HepG2 cells.

Next, the effects of uridine on O-linked glycosylation of FOXO1 were evaluated in HepG2 cells. FOXO1 is a transcription factor that mediates the effects of insulin on hepatic glucose production [18, 19]. O-linked glycosylation of FOXO1 promotes hepatic gluconeogenesis by activating the expression of gluconeogenic genes [20, 21]. HepG2 cells were transfected with a plasmid that expressed recombinant FOXO1 with a FLAG epitope. Purified recombinant FOXO1 was first detected with 1D Western blots using an anti-FLAG antibody, then with an antibody against O-GlcNAc. Recombinant FOXO1 exhibited substantially higher level of O-GlcNAc modification in HepG2 cells treated with uridine than control HepG2 cells (**Fig 1C**). Using 2D Western blot analysis of HepG2 total cell extracts with an anti-FLAG antibody, changes to the isoelectric point (pI) of glycosylated FOXO1 was examined. Compared to control HepG2 cells, an additional FLAG-positive protein spot was observed at a more basic pI value for HepG2 cells treated with uridine (**Fig 1D**). Treatment with a mixture of deglycosylases reduced the size of the protein spot with basic pI value and induced shifts toward acidic pI values. Both 1D and 2D Western blot analyses supported increases in O-linked glycosylation of FOXO1 following the treatment of HepG2 cells with uridine.

**Fig 2:**
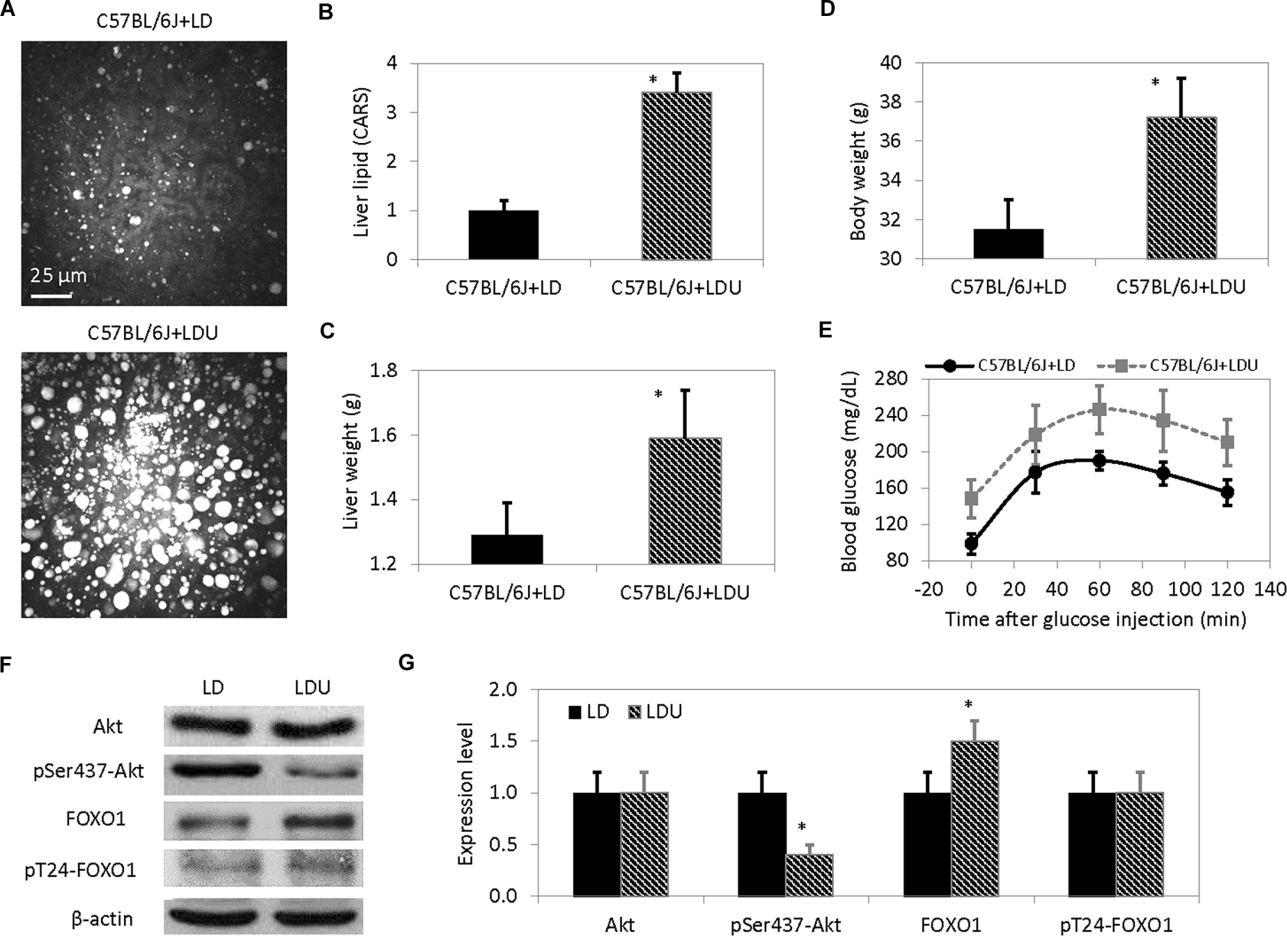
Chronic uridine administration induces liver lipid accumulation and elevated blood glucose level. (**A**) Assessment of liver lipid content with CARS microscopy. Images presented are 3D stacked of 30 frames at 1-micron increment along the vertical axis. (**B**) Quantitative analysis of average liver lipid content using CARS signal intensity. (**C**) Average liver weight and (**D**) body weight. (**E**) Blood glucose level as a function of time after glucose injection. Error bars are standard deviation values across 9 mice measured per animal group. (**F**) 1D Western blot analysis of liver Akt and FOXO1 expression and phosphorylation level. (**G**) Quantitative analysis of liver Akt and FOXO1 expression and phosphorylation level. Error bars are standard deviation across triplicate measurements. Asterisks indicate p-value <0.01 versus C57BL/6J+LD.

To evaluate the effects of long-term uridine administration, C57BL/6J mice were fed with a uridine-supplemented diet for 16 weeks. Assessment of liver lipid with CARS microscopy revealed tremendous increases in the accumulation of lipid droplets in mice fed with a uridine-supplemented diet compared to control mice (**Fig 2A**). On average, liver lipid content increased by more than three folds (**Fig 2B**) and both liver weight and body weight increased by approximately 20% in mice fed with a uridine-supplemented diet compared to control mice (**Fig 2C and 2D**). Following uridine-supplemented feeding, glucose tolerance tests revealed an elevated fasting blood glucose level and a reduced capability to remove blood glucose compared to control mice (**Fig 2E**). As anticipated, reduced phosphorylation of liver Akt was observed following uridine feeding (**Fig 2F**). While there was a slight increase in liver FOXO1 expression level, no change to FOXO1 phosphorylation level was observed (**Fig 2F and 2G**). Our data indicated that chronic uridine feeding induced fatty liver and reduced systemic glucose tolerance and liver insulin signaling.

**Fig 3:**
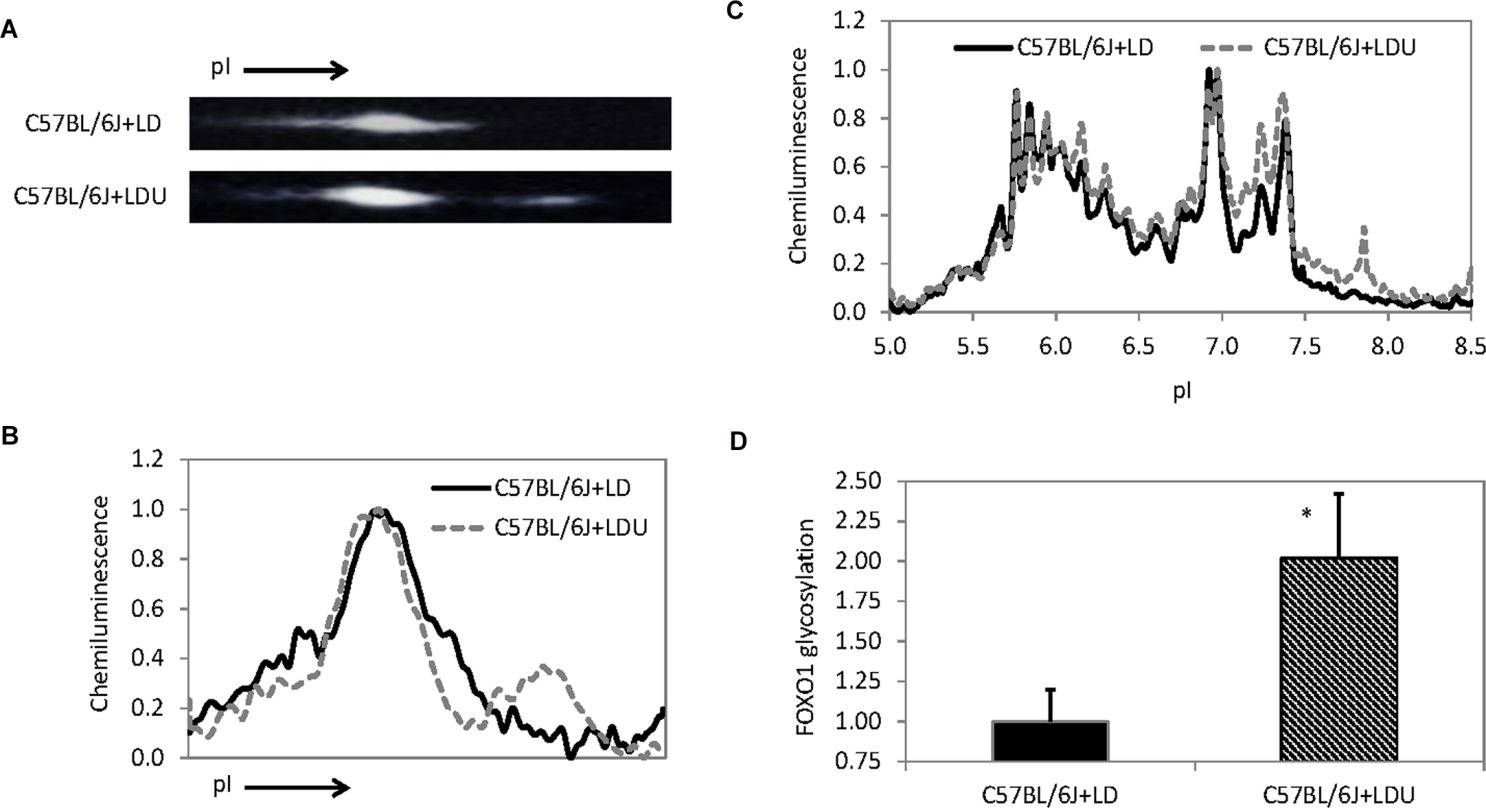
Glycosylation of FOXO1 detected with 2D Western blot and capillary isoelectric focusing (cIEF) immunoassay. (**A**) Total liver protein was evaluated with 2D WB using an antibody that recognizes FOXO1. (**B**) Chemluminescence intensity as a function of pI of the 2D WB shown in (**A**). Note an additional FOXO1 protein spot at high pI value was detected in the liver sample of C57BL/6J+LDU mice but not in C57BL/6J+LD mice. (**C**) Liver FOXO1 distribution as a function of pI detected with cIEF immunoassay. Peak chemiluminescence was normalized to 1. Peaks from pI 7.5 to 8.5 are due to FOXO1 glycosylation in the liver tissue of C57BL/6J+LDU mice. (**D**) Quantitative area under the curve analysis (pI 7.5–8.5) of glycosylated FOXO1 isoform as a function of liver tissues. Error bars are standard deviation values across 9 measurements. Asterisk indicates p-value < 0.01 versus C57BL/6J+LD.

Interestingly, 2D Western blot analysis of total liver extracts with FOXO1 antibody revealed an additional protein spot at a more basic pI value in mice fed with a uridine-supplemented diet compared to control mice (**Fig 3A and 3B**). The presence of the FOXO1 protein spot at a higher pI value indicated that FOXO1 was glycosylated. Like 2D Western blot, cIEF immunoassays revealed additional peaks in the pI 7.5–8.5 range in the liver tissues of mice fed with a uridine-supplemented diet, which were absent in the liver tissues of control mice (**Fig 3C**). Thus, cIEF immunoassays could serve as a rapid means to evaluate glycosylation state of FOXO1 by using the integrated chemiluminescence signal from pI 7.5–8.5 (**Fig 3D**).

**Fig 4:**
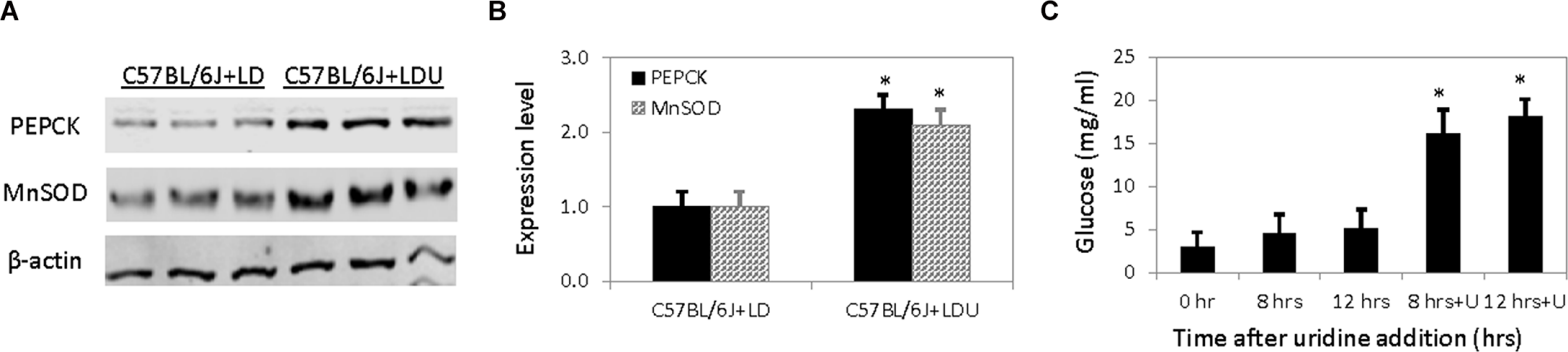
Uridine stimulates hepatic gluconeogenesis. (**A**) Increased expression of liver PEPCK and MnSOD following chronic uridine feeding. (**B**) Quantitative analysis of Western blot data on protein expression level. (**C**) Glucose production a function of time after uridine addition in HepG2 cells. Error bars are standard deviation values across three mice per animal group or triplicated HepG2 cell cultures. Asterisks indicate p-value <0.01 versus control.

FOXO1 glycosylation is generally associated with elevated expression of hepatic gluconeogenic and stress response genes such as phosphoenolpyruvate carboxykinase and manganese superoxide dismutase, respectively [20, 21]. To evaluate the effects of FOXO1 glycosylation, 1D Western blots were employed to measure the expression levels of liver PEPCK and MnSOD. As anticipated, the expression levels of both PEPCK and MnSOD increased by more than two folds in mice fed with a uridine-supplemented diet compared to control mice (**Fig 4A and 4B**). Increased expression of PEPCK is associated with increased glucose production [22]. To determine if uridine promotes hepatic glucose production, we measured glucose concentration in HepG2 cells treated with uridine. On average, uridine treatment increased glucose concentration in HepG2 cells by over three folds (**Fig 4C**). It is plausible that uridine promotes hepatic glucose production via FOXO1 glycosylation.

**Fig 5:**
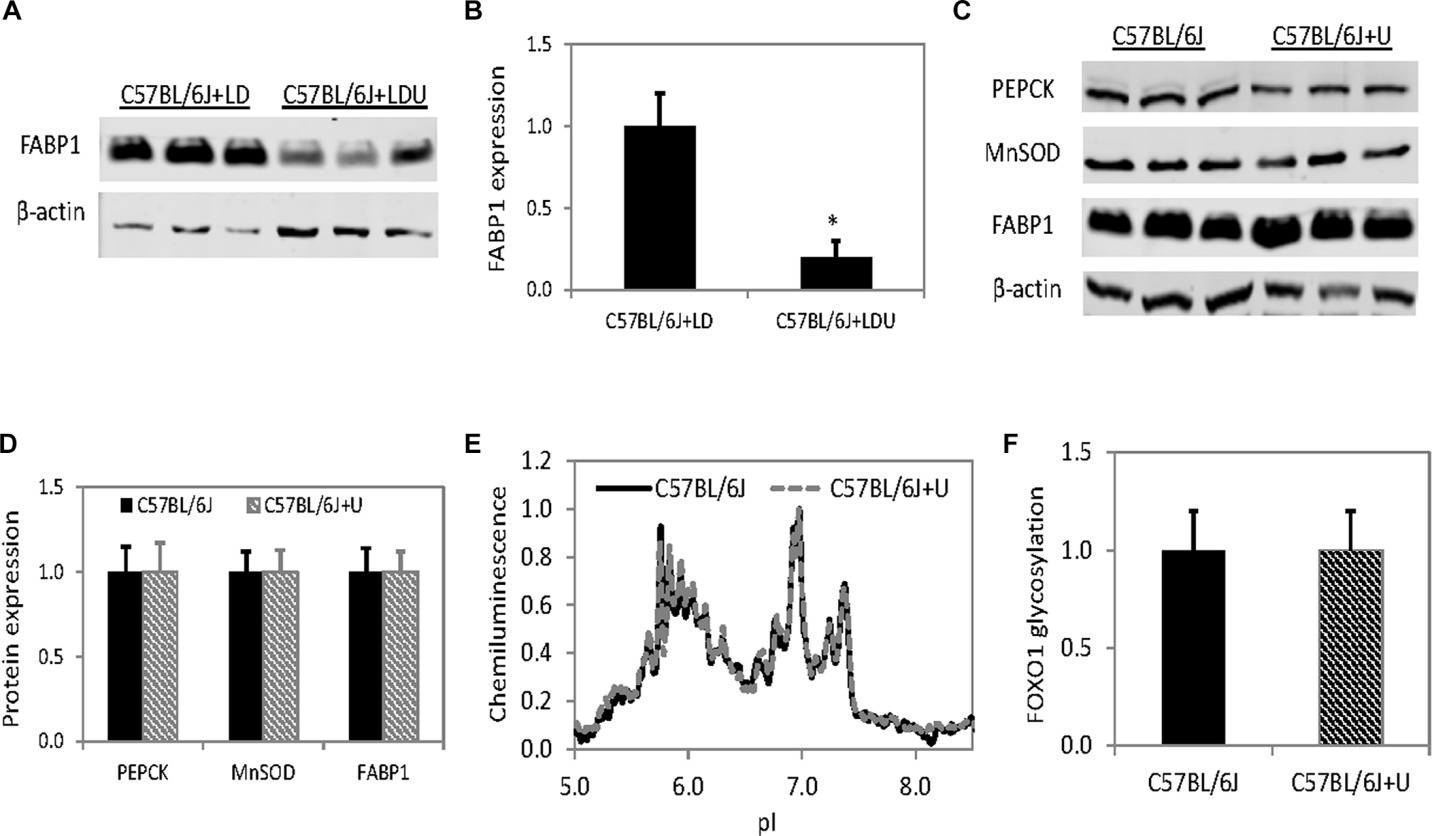
Differential short- and long-term effects of uridine on protein expression level. (**A**) Reduced expression of FABP1 following chronic uridine feeding. (**B**) Quantitative analysis of Western blot data on FABP1 expression level. (**C**) Expression level of PEPCK, MnSOD, and FABP1 are unchanged following 5 days of feeding with uridine supplemented diet. (**D**) Quantitative analysis of Western blot data on protein expression level in (**C**). Error bars are standard deviation values across three mice per animal group. Asterisks indicate p-value <0.01 versus control. (E) Liver FOXO1 distribution as a function of pI detected with cIEF immunoassay. Peak chemiluminescence was normalized to 1. (**F**) Quantitative area under the curve analysis (pI 7.5–8.5) of glycosylated FOXO1 isoform as a function of liver tissues. Error bars are standard deviation values across 9 measurements.

In addition to changes to hepatic gluconeogenesis, chronic uridine feeding also led to a 5-fold suppression in the expression level of liver specific fatty acid binding protein FABP1 (**Fig 5A and 5B**). How chronic uridine treatment repressed FABP1 expression was unknown. However, the expression and phosphorylation of other member of the fatty acid binding proteins, such as FABP4 in adipocytes, was stimulated by insulin [23, 24]. Reduced liver insulin signaling activity following uridine treatment could suppress the expression of FABP1. Repression of FABP1 expression was associated with fatty liver disease in both mice and humans [25, 26] and could be a contributing factor to the development of fatty liver in mice chronically fed with uridine.

The effects of uridine feeding on liver lipid and glucose metabolism were dependent on the duration of treatment. Within 5 days of uridine feeding, the impacts on insulin signaling, systemic glucose tolerance, and protein glycosylation were already observed [15]. However, the effects of uridine on the expression levels of PEPCK, MnSOD, and FABP1 were not seen during the same time frame (**Fig 5C and 5D**). Furthermore, no glycosylation of FOXO1 protein was detected with cIEF immunoassays (**Fig 5E and 5F**). After 5 days of uridine feeding, no liver lipid droplet accumulation was observed and the free fatty acid and triacylglyceride profiles were nearly identical for mice fed with a uridine-supplemented diet compared to control mice [27]. Thus, the manifestation of uridine’s impacts on liver lipid accumulation and expression of gluconeogenic genes was only observable in mice with chronic uridine feeding.

## Discussion

In this study, we report chronic uridine feeding induced liver lipid accumulation and pre-diabetic conditions in mice. Uridine feeding caused severe hepatic steatosis and significant liver and body weight gain. Uridine feeding also increased fasting blood glucose level and impaired blood glucose clearance following glucose injection. On one hand, uridine feeding suppressed liver insulin signaling activity and the expression of FABP1. On the other hand, uridine feeding promoted O-linked glycosylation of FOXO1 and expression of liver gluconeogenic genes. The effects of long-term uridine treatment on liver lipid and glucose metabolism and systemic glucose tolerance should be considered carefully when evaluating the therapeutic potentials of uridine.

Differential short- and long-term effects of uridine on liver lipid accumulation and expression level of liver proteins could be due to homeostatic control of circulating uridine level. Uridine is the only significant pyrimidine nucleoside in the circulation [28]. Circulating uridine is essentially degraded in a single pass by the liver and replaced with *de novo* biosynthesis [29]. The manifestation of uridine effects on the liver could be delayed due to low bioavailability of uridine in the circulation [30]. In contrast, the full effects of uridine were seen within 48 hours of treatment in cultured HepG2 cells, where high concentration of uridine was sustained.

The effects of uridine on liver protein glycosylation and gluconeogenesis appear to be non-specific and mirror those of glucosamine. The synthesis of UDP-GlcNAc, a donor substrate for protein glycosylation, requires both UTP and N-acetylglucosamine. Phosphorylation of uridine generates UTP and an amide bond between acetic acid and glucosamine generates N-acetylglucosamine. Uridine treatment in both mice and HepG2 cells non-specifically increased the overall protein glycosylation profiles. Proteomics data revealed a wide range of proteins with different cellular functions and locations were affected by uridine treatment [15]. Both glucosamine and uridine induced FOXO1 glycosylation and stimulated *de novo* gluconeogenesis in HepG2 cells [21]. The combinatorial treatment of uridine and glucosamine was linked to increased glycosylation of a glucose transported GLUT4 and reduced insulin-stimulated glucose transport in skeletal muscle [12]. It is clear that both uridine and glucosamine serve complementary function with regard to UDP-GlcNAc availability for protein glycosylation.

The precise mechanism underlying the correlation between uridine and insulin resistance remains to be investigated. The combined effects of reduced liver insulin signaling activity and increased gluconeogenesis clearly contributed to the increase in blood glucose level. However, it is unclear how uridine suppressed insulin signaling activity. A plausible mechanism is via the cross-talk between protein glycosylation and phosphorylation [31]. By promoting protein glycosylation, uridine could interfere with the phosphorylation of insulin-regulated proteins. Indeed, increased glycosylation of GLUT4 and FOXO1 was observed together with reduced insulin-stimulated glucose transport and the suppression of hepatic gluconeogenesis, respectively [12, 20, 21]. O-linked glycosylation of insulin receptor substrate 1 (IRS1) is also a known mechanism that regulates IRS phosphorylation [32]. Purines and pyrimidines complement one another in nucleic acid base pairing. ATP participates in protein phosphorylation and UTP participates in protein glycosylation. The cross-talk between protein phosphorylation and glycosylation highlights additional purine-pyrimidine complementarity that extends beyond the basic structure of the genetic code.

Uridine has opposing effects on liver lipid metabolism. This study revealed that uridine induced fatty liver when administered chronically by itself. However, uridine prevents drug-induced fatty liver when co-administered with other drugs. A common acting mechanism of uridine is via restoration of liver homeostasis perturbed by drugs administration. For example, uridine co-administration restored the pyrimidine nucleotide pool, which was depleted due to the use of zalcitabine [5]. Uridine co-administration suppressed liver protein hyperacetylation due to the use of fenofibrate [6]. When co-administered with tamoxifen, uridine diverted diacylglyceride and triacylglyceride into membrane phospholipids biosynthesis and reduced cytoplasmic lipid droplet accumulation [7]. Fatty liver is a manifestation of hepatotoxicity induced by many commonly used drugs with diverse acting mechanisms [33]. The versatile restorative capability of uridine suggests that it could serve as an effective co-drug to prevent drug-induced fatty liver.

Uridine concentration could potentially be elevated in a liver specific manner. In vertebrates, two homologs of uridine phosphorylase (UPase) are present, UPase1 and UPase2 [34]. Uridine phosphorylase is an enzyme that catalyzes the reversible conversion of uridine to uracil. Inhibition of enzymatic activity of UPases with 5-benzylacyclouridine or genetic knock-out of UPase1 gene both resulted in elevated circulating and tissue uridine concentration [35, 36]. UPase1 and UPase2 have ~62% sequence identity and same catalytic activity [34]. However, UPase1 is ubiquitously present in every tissue while UPase2 is a liver-specific protein [34]. Furthermore, the active site of UPase2 has a disulfide bridge between two cysteine residues, which is absent in UPase1 [37]. Consequently, enzymatic activity of UPase2, but not UPase1, is sensitive to redox regulation. Catalytic activity of UPase2 is active in reduced state with the disulfide bridge and inactive in oxidized state without the disulfide bridge. The structural difference in the active sites of UPase1 and UPase2 suggests that selective inhibitor of UPase2 could be rationally designed. Increasing endogenous liver uridine concentration via inhibition of UPase2 could protect the liver against drug-induced lipid accumulation with minimal systemic effects on other tissues. Like many other therapeutic molecules, uridine has its own unintended consequences on the biological systems. Further characterization of the side effects and careful design of the intervention strategies should maximize the therapeutic potentials of uridine.
